# Copper nanomaterial antimicrobial coatings: ion release, transparency, and wash durability across biomedical, textile, and packaging applications

**DOI:** 10.3389/fchem.2026.1949389

**Published:** 2026-09-16

**Authors:** Nurhidayatul Warda Muhamad Shuhaimi, Jei Vincent, Kam Sheng Lau, Siew Xian Chin, Shahariar Chowdhury, Theerakamol Pengsakul, Chin Hua Chia

**Affiliations:** 1 Materials Science Program, Department of Applied Physics, Faculty of Science and Technology, Universiti Kebangsaan Malaysia, Bangi, Selangor, Malaysia; 2 ASASIpintar Program, Pusat PERMATA@pintar Negara, Universiti Kebangsaan Malaysia, Bangi, Selangor, Malaysia; 3 Quantum Materials and Technology Research Group, Universiti Kebangsaan Malaysia, Bangi, Selangor, Malaysia; 4 Faculty of Environmental Management, Prince of Songkla University, Hatyai, Songkhla, Thailand; 5 Health and Environmental Research Center (HERC), Faculty of Environmental Management, Prince of Songkla University, Hatyai, Songkhla, Thailand

**Keywords:** antimicrobial coatings, copper nanomaterials, durability, ion release, optical transparency

## Abstract

Healthcare-associated infections remain a major burden on health systems, with 8.9 million episodes estimated annually across EU/EEA hospitals and long-term care facilities and approximately 518,000 in US acute care hospitals in 2023, driving demand for persistent antimicrobial surface coatings. Copper nanomaterials (CuNMs) offer multi-mechanism bactericidal and virucidal activity, including membrane disruption, reactive oxygen species generation, and photothermal hyperthermia, at an approximately 150-fold lower feedstock cost than silver, yet commercial deployment remains limited. This review synthesises 498 Scopus-indexed articles (2015–2026) on CuNM antimicrobial coatings to identify the structural barrier to translation. A critical trilemma emerges: no published system simultaneously achieves optical transmittance >80% at 550 nm, ≥2-log bacterial reduction, and durability over ≥50 home launderings or ≥3 months continuous efficacy. The five principal CuNM forms, Cu^0^, Cu_2_O, CuO, CuS, and Cu–MOFs, occupy distinct niches in the efficacy–transparency–stability design space. Copper sulfide nanoparticles and sub-0.5 wt% Cu-MOFs are the most promising candidates for transparent applications. Polydopamine anchoring establishes a wash-durability benchmark of 40 laundering cycles at 90% efficacy retention. Stimulus-responsive pH- and NIR-triggered release systems extend functional lifetime while limiting cytotoxic overexposure. An 8-parameter Minimum Reporting Standard is proposed to resolve heterogeneous reporting that currently impedes cross-study comparison, regulatory assessment, and commercial investment.

## 1 Introduction

Healthcare-associated infections (HAIs) remain a major burden on health systems. European point prevalence surveys estimate 8.9 million episodes annually across EU/EEA hospitals and long-term care facilities, and the most recent ECDC survey reports 4.3 million hospital patients acquiring at least one HAI each year (European Centre for Disease Prevention and Control, 2024); in the United States, the Centers for Disease Control and Prevention estimated approximately 518,000 HAIs in acute care hospitals in 2023. Direct costs exceed USD 45 billion per year (Centers for Disease Control and Prevention, 2024). Contaminated environmental surfaces, bed rails, doorknobs, touchscreens, and high-touch corridor surfaces, serve as persistent reservoirs for methicillin-resistant *Staphylococcus aureus* (MRSA), vancomycin-resistant *Enterococcus* (VRE), and multidrug-resistant Gram-negative organisms. Current episodic decontamination practices, alcohol wipes, hypochlorite solutions, and hydrogen peroxide vapour, provide no residual protection between cleaning events, and quaternary ammonium compound (QAC) coatings are increasingly compromised by documented resistance in clinical strains. A new generation of persistent, self-sustaining broad-spectrum antimicrobial surface technologies is urgently needed (Singh et al., 2023).

Copper has been exploited for antimicrobial purposes since antiquity, and copper alloy surfaces received landmark EPA registration under FIFRA in 2008, the first, and still the only, solid material to receive such regulatory recognition for continuous antimicrobial activity. That precedent sets the benchmark against which CuNM coatings must be judged: 490 alloys are registered at a minimum of 60 wt% copper, and clinical evaluation across three intensive care units led Schmidt et al. to report a 96.8% reduction in combined MRSA and VRE surface burden and Salgado et al. a 58% reduction in healthcare-associated infection incidence (Salgado et al., 2013; Schmidt et al., 2012). Bulk alloys also possess an intrinsic durability advantage that no coating can replicate, since abrasion exposes fresh copper rather than removing the active phase, and they operate at full technology readiness. Copper-doped glasses occupy a narrower position again, concentrated in biomedical fillers such as dental composites, polymethyl methacrylate bone cements and borate bioactive glasses for bone and wound healing, where burst-then-sustained Cu release is advantageous but brittleness precludes large-area surface application. Set against these alternatives, the case for CuNM coatings rests on four bounded advantages rather than on general superiority. First, coatings can be applied to existing glass, polymer, textile and ceramic surfaces, whereas an alloy solution requires replacing the object, and the majority of high-touch area in a clinical environment is neither metallic nor readily replaceable. Second, the mass efficiency is two to three orders of magnitude greater: a copper alloy fitting carries kilograms of copper, whereas a textile finish at 1 wt% on 150 g/m^2^ fabric carries 1.5 g Cu/m^2^. Third, optical transparency is structurally unattainable in a 60 wt% copper alloy and achievable in principle for CuNM systems, the sole axis on which alloys cannot compete. Fourth, stimulus-responsive and reservoir-based release architectures have no counterpart in a bulk material. One regulatory asymmetry qualifies these advantages: under the EU Biocidal Products Regulation nanoforms must be assessed separately from bulk copper, so CuNM coatings cannot inherit the alloy precedent. Studies in which copper functioned purely as a bulk alloy component were accordingly excluded from this corpus, a deliberate scope decision rather than an oversight. Transitioning from bulk copper alloy to copper nanomaterials (CuNMs) unlocks qualitatively new functionality: dramatically increased surface area-to-volume ratios, tunable ion release kinetics controllable through particle size, surface chemistry, and matrix architecture, and photophysical properties, including localised surface plasmon resonance (LSPR) and photothermal activity, entirely absent in bulk metal. At current metal prices (USD 13–14/kg for copper against approximately USD 2,100/kg for silver, August 2026) (London Bullion Market Association, 2026; London Metal Exchange, 2026), copper provides an approximately 150-fold feedstock cost advantage over silver, and its multiple simultaneous killing mechanisms (Cu^2+^ membrane disruption, reactive oxygen species generation, and photothermal hyperthermia) make resistance development intrinsically unlikely, a decisive advantage over single-mechanism antimicrobial agents (Khalifa et al., 2025; Pellosi et al., 2025).

Despite this favourable profile, commercial deployment of CuNM antimicrobial coatings has been remarkably slow. A structured survey of 498 corpus papers, assembled by a Scopus search targeting copper nanomaterial coating systems with confirmed surface-contact antimicrobial outcomes, reveals a structural explanation: the three properties most critical for commercial viability have been studied almost entirely in isolation. (i) Antimicrobial efficacy (≥99%, or 2-log, bacterial reduction under standardised contact conditions), (ii) optical transparency (≥80% luminous transmittance at 550 nm with haze below 30%), and (iii) wash and operational durability (maintenance of ≥99% bacterial reduction through ≥50 home launderings, equivalent to approximately ten AATCC 61-2A cycles, or ≥3 months of continuous surface deployment) are each optimised in separate, largely non-overlapping literature streams. These three criteria differ in provenance, and we state this explicitly. The efficacy criterion is not our own: ≥99% (2-log) reduction is the pass mark (antibacterial activity value R ≥ 2.0) specified by ISO 22196:2011 and JIS Z 2801 for non-porous surfaces, with ISO 21702:2019 the corresponding antiviral method. It is also permissive relative to the route by which copper surfaces are actually registered in the United States: EPA method MLB SOP MB-41 (Environmental Protection Agency, 2025) requires ≥99.9% (3-log) reduction of bacteria and viruses within one to 2 hours, and the accompanying residual self-sanitizing protocol imposes six wet and dry wear cycles with re-inoculation before the final efficacy test, so coatings meeting the criterion adopted here may still fail US public-health registration. The transparency and durability criteria, by contrast, are our own operational definitions. No standard specifies a transmittance threshold for antimicrobial coatings; ≥80% at 550 nm is the *de facto* baseline of the transparent conductive film industry, benchmarked to indium tin oxide, and we pair it with a haze limit because transmittance alone does not capture the milky appearance produced by scattering (both measurable by ASTM D1003). Similarly, ASTM E3162-18 and AATCC 61 define laundering durability methods but set no pass threshold, so the 50-laundering criterion corresponds to the highest of the three durability tiers (5, 25 and 50 washes) recognised in ASTM E3162-18, noting that the practice defines those tiers rather than setting a pass criterion. No stakeholder or industry consultation informed these two values, and they should be read accordingly. The consequence is a durability–transparency–efficacy trilemma: no published CuNM coating system simultaneously satisfies all three criteria. The trilemma is not, however, an artefact of these particular values. Of the three corpus articles reporting a quantitative visible transmittance (Supplementary Dataset S1, Sheet 4), tightening the optical criterion to 90% leaves only Ren et al. (2018) and relaxing it to 70% additionally admits Jampa et al. (2022) (Jampa et al., 2022; Ren et al., 2018); yet none of the eight articles treating transparency as a design objective reports a quantitative contact-kill log reduction together with a standardised durability test at any threshold. The conclusion is therefore robust to the choice of criteria, which constrain how many systems approach the viable zone but not whether any reaches it. This review synthesises the full corpus evidence to map the trilemma, quantify current performance limits across each dimension, and chart a concrete research agenda for its resolution (Khalifa et al., 2025; Pellosi et al., 2025).

The 498-article corpus was assembled through a structured SCOPUS search combining controlled vocabulary terms for copper nanomaterial coating systems with confirmed antimicrobial outcomes, spanning 2015–2026 with peak publication density in 2022–2025. Articles were excluded where copper functioned purely as a bulk alloy component, where antimicrobial testing was conducted only in solution rather than surface-contact format, or where no quantitative efficacy metric was reported. The complete search specification, the screening cascade from 629 records returned to the 498 retained, the full corpus listing, and the screening rules applied to every quantitative statement in this review are provided as Supplementary Dataset S1, with the underlying Scopus export deposited as Supplementary Data S2. The 498 retained articles were classified across 11 thematic categories (Figure 1). As Figure 1 illustrates, the corpus is dominated by Textile and Wearable Coatings (CAT6, 200 articles, 40.2%) and Synthesis and Characterisation (CAT1, 108 articles, 21.7%), reflecting both the maturity of functional textile finishing technology and the research community’s strong foundation in nanomaterial synthesis. In marked contrast, Transparent and Scalable Coatings (CAT9, 1 article) and Toxicity and Safety (CAT10, 6 articles) are the two smallest categories, together comprising fewer than 1.5% of the corpus. This structural misalignment is critical: CAT9 and CAT10 represent precisely the domains most essential to commercial deployment of CuNM coatings on high-touch transparent surfaces, and their near-absence from the literature underscores the field’s systematic under-investment in translation-critical properties. Full texts were retrievable for 303 of the 498 papers (60.8%), and every corpus statistic reported in this review states this denominator explicitly. Only eight papers treat optical transparency of the coating as a stated design objective, of which three report a quantitative visible transmittance value; 15 of 303 (5.0%) report any environmental fate or ecotoxicity endpoint, and none reports a complete life-cycle assessment. The full corpus, the screening cascade, the curated evidence tables underlying each of these counts, and the screening rules themselves are provided as Supplementary Dataset S1. These figures confirm that the corpus’s commercial blind spots are deep and consistent (Khalifa et al., 2025; Pellosi et al., 2025; Singh et al., 2023).

**FIGURE 1:**
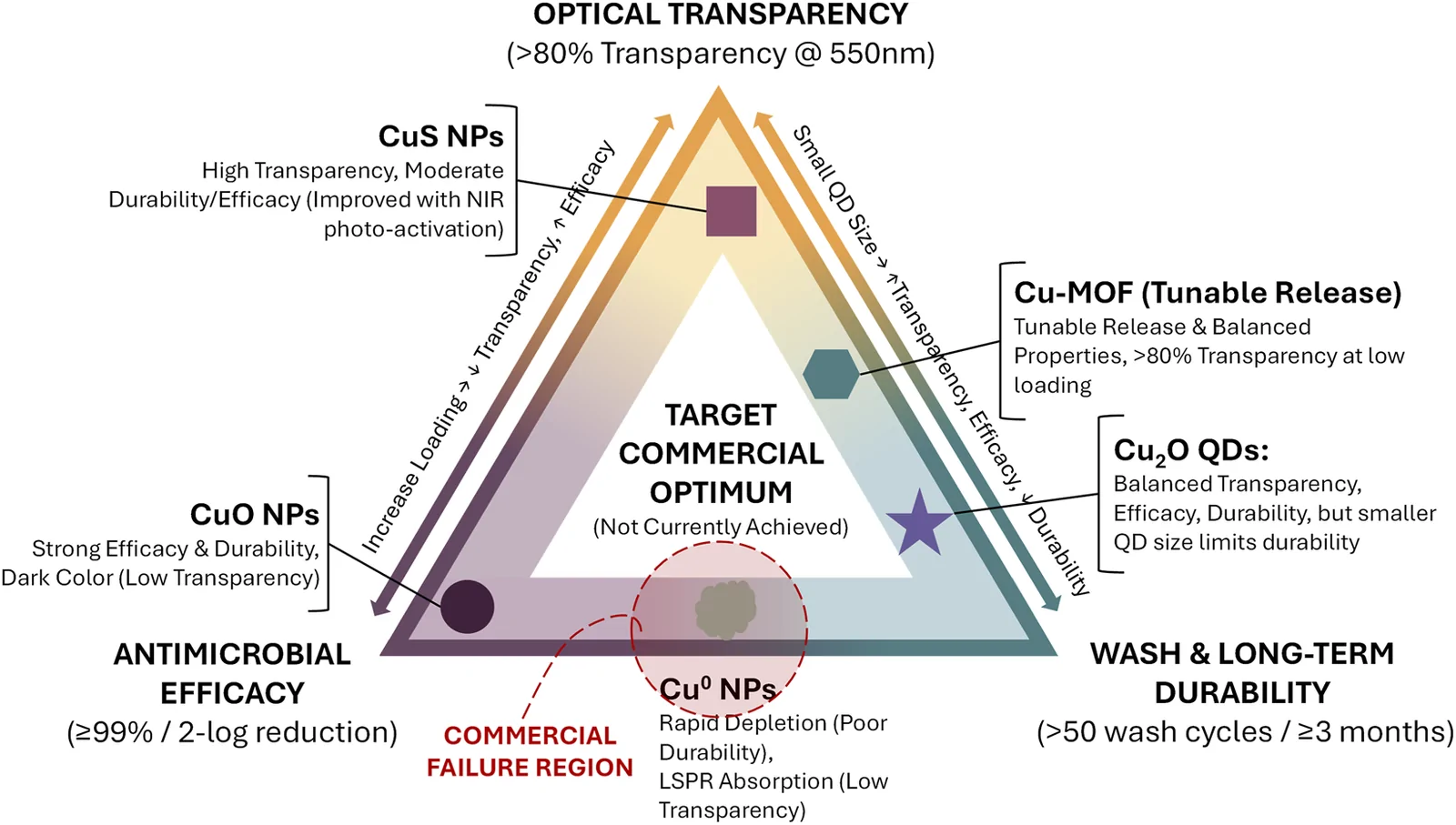
Distribution of the 498-article Scopus corpus (2015–2026) across 11 thematic categories, coloured by relevance to review scope. Background (grey): underpins synthesis knowledge; Core (blue): integral to coating design; Core (primary) (red): directly addresses the durability–transparency–efficacy trilemma; Essential (teal): regulatory/safety evidence; Synthesis/context (purple): reference reviews. CAT6 Textile and Wearable Coatings (n = 200, 40.2%) and CAT1 Synthesis & Characterisation (n = 108, 21.7%) dominate the corpus, reflecting the maturity of functional textile finishing and nanomaterial synthesis research. CAT9 Transparent & Scalable Coatings (n = 1) and CAT10 Toxicity & Safety (n = 6) are critically under-represented, together less than 1.5% of the corpus, despite being the two domains most essential to commercial deployment of CuNM coatings on high-touch transparent surfaces. Total articles: N = 498.

## 2 Copper nanomaterial forms, properties, and coating compatibility

Five principal CuNM forms populate the antimicrobial coating literature, each occupying a distinct niche in the efficacy–transparency–stability design space.

### 2.1 Metallic copper nanoparticles (Cu0 NPs)

Metallic copper nanoparticles (Cu^0^ NPs) provide rapid initial antibacterial activity through surface oxidation to Cu^2+^ in ambient air, exhibit characteristic orange-red localised surface plasmon resonance (LSPR) absorption at 570–590 nm, and are the lowest-cost form; their primary limitation is susceptibility to agglomeration without stabilising agents (PVP, citrate) and fast depletion of the surface oxide layer (Glazkova et al., 2022; Khalifa et al., 2025).

### 2.2 Cuprous oxide (Cu_2_O) NPs

Cuprous oxide (Cu_2_O) NPs are p-type semiconductors (bandgap 2.17 eV) with moderate visible absorption and visible-light photocatalytic activity; their solubility rises sharply below pH 6, providing inherent infection-responsiveness, and gram-scale synthesis by chemical reduction has been demonstrated (Dam et al., 2024; Nayak et al., 2025; Rai, 2023).

### 2.3 Cupric oxide (CuO) NPs

Cupric oxide (CuO) NPs represent the thermodynamically stable form, offering the slowest baseline Cu^2+^ release, suited to sustained-release architectures, but their broad d-d and charge-transfer absorption across the entire visible range (dark brown-black coloration) makes them fundamentally incompatible with transparent coating applications (Glazkova et al., 2022; Okoye et al., 2023; Tavakoli et al., 2019).

### 2.4 Copper sulfide (CuS) NPs

Copper sulfide (CuS) NPs absorb primarily in the NIR (900–1100 nm), imposing only a faint visible tint (Bose et al., 2025; Sun et al., 2024), and generating photothermal hyperthermia (>50 °C) under 808 or 1064 nm irradiation (Qiao et al., 2023; Yan et al., 2024), the defining advantage for transparent antimicrobial coatings where optical properties must be maintained (Gao et al., 2025).

### 2.5 Copper metal-organic frameworks (Cu-MOFs)

2.5 Copper metal-organic frameworks (Cu-MOFs), exemplified by Cu-BTC (HKUST-1, BET surface area ~1500–2000 m^2^/g), release Cu^2+^ through combined framework dissolution and pore-diffusion pathways whose kinetics are directly engineered through linker chemistry and pore geometry (Hubab et al., 2025; Kang et al., 2025), offering the most designable release platform; teal-blue coloration at high loadings diminishes to acceptable levels below ~0.5 wt% (Alhussaini et al., 2026; Men et al., 2025; Zheng et al., 2022). Table 1 compares these five forms across the properties most relevant to coating design.

**TABLE 1:** Comparative properties of the five principal CuNM forms relevant to antimicrobial coating design.

Property	Cu^0^ NPs	Cu_2_O NPs	CuO NPs	CuS NPs	Cu-MOF
Visible colour	Orange-red	Yellow-orange	Dark brown-black	Near-colourless	Teal-blue
Transparency potential	Low	Moderate	Very low	High	High (<0.5 wt%)
Ion release rate	Fast (burst)	Moderate, pH-sensitive	Slow-sustained	Slow + NIR-triggered	Tunable (pore-designed)
Oxidative stability	Low	Moderate	High	High	Moderate-high
Photocatalytic activity	None	Visible-light	Weak	NIR photothermal	Linker-dependent
Synthesis scalability	High	High	High	Moderate	Moderate-low
Key references	Batenkova et al. (2026), Khalifa et al. (2025), Pellosi et al. (2025), Singh et al. (2023)	Bi et al. (2026), Dam et al. (2024), Nayak et al. (2025), Rai (2023), Ran et al. (2021)	Glazkova et al. (2022), Okoye et al. (2023), Tavakoli et al. (2019)	Bose et al. (2025), Qiao et al. (2023), Sun et al. (2024), Yan et al. (2024)	Hubab et al. (2025), Kang et al. (2025), Men et al. (2025)

Properties compared: visible colour, transparency potential, ion release rate, oxidative stability, photocatalytic activity, and synthesis scalability. CuNM forms covered: Cu0 NPs, Cu2O NPs, CuO NPs, CuS NPs, and Cu-MOF. Key supporting references per form are listed in the bottom row.

The antimicrobial killing mechanisms of CuNMs operate through multiple simultaneous pathways whose relative contributions differ by species and target organism (Figure 2). Cu^2+^ ions displace essential Ca^2+^ and Mg^2+^ from bacterial membrane phospholipids through charge-based competition, disrupting membrane integrity and triggering osmotic lysis at concentrations of 0.5–2 mg/L for susceptible Gram-positive organisms (Khalifa et al., 2025; Okoye et al., 2023). Intracellular Cu^2+^ catalyses hydroxyl radical production via Fenton-like chemistry (Cu^+^ + H_2_O_2_ → Cu^2+^ + OH^•^ + OH^−^), targeting DNA strand breaks, lipid peroxidation, and enzyme inactivation in sequence (Okoye et al., 2023). Gram-negative bacteria show 4–10-fold higher MIC values compared to Gram-positive equivalents, reflecting the protective role of the outer membrane LPS layer as a Cu^2+^ diffusion barrier, a key reason why coatings must be benchmarked against both organism classes rather than *S. aureus* alone (Khalifa et al., 2025; Pellosi et al., 2025). CuS NPs add photothermal hyperthermia (>50 °C under NIR irradiation) as a mechanistically independent third killing pathway, while Cu-MOF frameworks contribute visible-light photocatalytic ROS generation (Bose et al., 2025; Hubab et al., 2025). This mechanistic redundancy is the primary evolutionary argument against Cu resistance: no single-point mutation can simultaneously confer tolerance to membrane disruption, Fenton-based ROS, photothermal stress, and Cu^2+^ ion toxicity. No clinical CuNM resistance has been documented despite decades of copper alloy surface deployment, validating this mechanistic diversity as a durable antimicrobial principle (Foffa et al., 2022; Pellosi et al., 2025).

**FIGURE 2:**
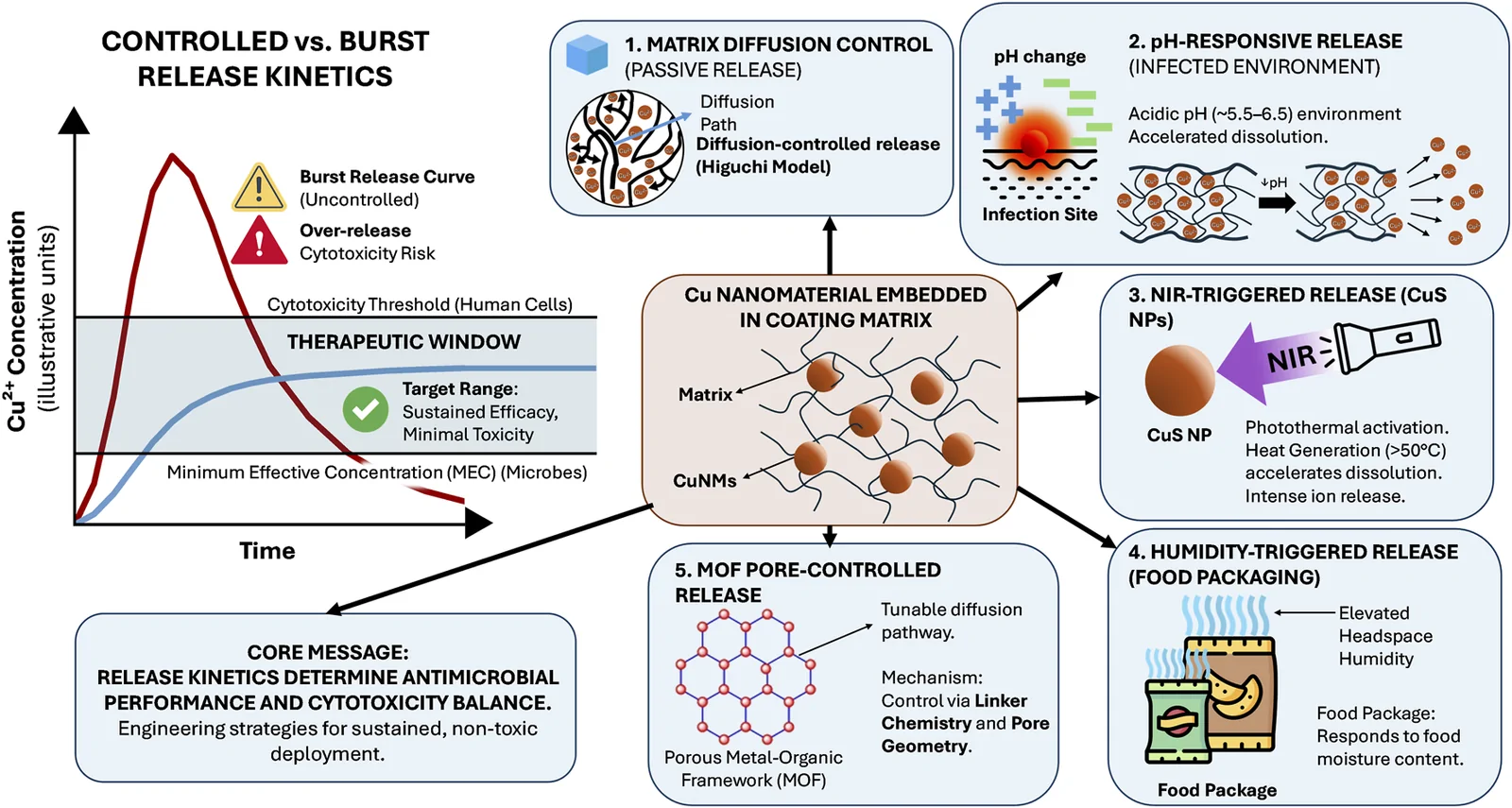
Multi-mechanism antibacterial pathways of copper nanomaterials. Cu^2+^ ions disrupt bacterial membrane integrity through competitive displacement of Ca^2+^/Mg^2+^ from phospholipids, and drive intracellular Fenton-like hydroxyl radical (OH•) production targeting DNA strand breaks, lipid peroxidation, and enzyme inactivation. CuS nanoparticles add NIR-activated photothermal hyperthermia (>50 °C) as a mechanistically independent third killing mode; Cu-MOF frameworks contribute visible-light photocatalytic reactive oxygen species (ROS) generation. Mechanistic redundancy across these four non-overlapping pathways underlies the absence of documented clinical CuNM resistance despite decades of copper alloy surface deployment.

A critical and underappreciated design factor is the oxidation state cascade: Cu^0^→Cu_2_O→CuO progresses continuously under ambient air and humidity, altering both ion release kinetics and optical properties over the coating’s operational lifetime. Cu^0^ NPs <20 nm are substantially surface-oxidised within hours of deposition; the resulting Cu^0^@Cu_2_O/CuO core-shell architecture produces an initial burst-release phase followed by diffusion-limited slower release as the oxide shell thickens (Bi et al., 2026; Khalifa et al., 2025; Ran et al., 2021). For coatings requiring long-term optical stability, oxidation state drift toward the strongly absorbing CuO form directly degrades transparency (Batenkova et al., 2026). Time-resolved XPS characterisation of Cu speciation at multiple time points throughout operational lifetime, reported in only a small minority of corpus studies, is a minimum requirement for credible durability claims. Surface functionality and particle size also directly govern coating performance: below ~10 nm, quantum confinement reduces visible absorption in Cu_2_O NPs and accelerates dissolution kinetics (Dam et al., 2024); bifunctional anchoring ligands (catechol-amine compounds) must stabilise NPs against agglomeration while simultaneously forming substrate bonds, requirements that are frequently in tension and that drive the anchoring chemistry strategies described in Section 5 (Durability) (Glazkova et al., 2022; Hu et al., 2022; Ran et al., 2021).

## 3 Coating fabrication strategies

The fabrication method is the primary determinant of adhesion strength (setting the durability ceiling), nanoparticle dispersion quality (governing efficacy uniformity), and deposition throughput (determining scalability), three properties that current methods cannot simultaneously maximise (Figure 3; Khalifa et al., 2025; Singh et al., 2023).

**FIGURE 3:**
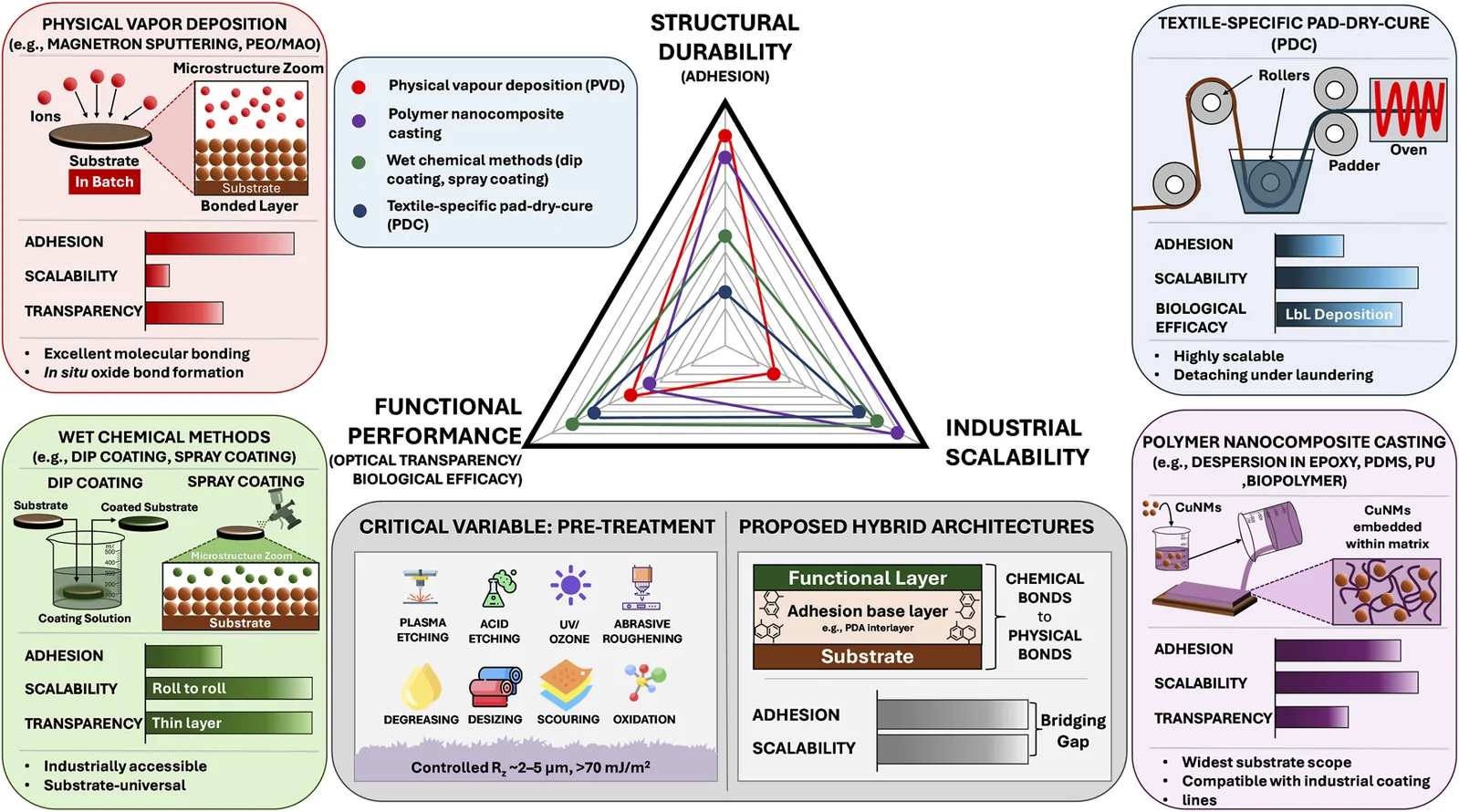
Structure–property relationships in engineered CuNM coating architectures. The fabrication method simultaneously governs adhesion strength (durability ceiling), nanoparticle dispersion homogeneity (efficacy uniformity), and deposition throughput (scalability), three performance ceilings no single method can simultaneously maximise. High-adhesion vacuum and electrolytic methods (magnetron sputtering, PEO/MAO) are batch-limited to specific substrates, whereas roll-to-roll-compatible wet chemical and textile pad-dry-cure methods require additional anchoring chemistry (e.g., polydopamine interlayer) to attain competitive wash durability. Hybrid architectures coupling a high-adhesion base layer with a scalable functional deposition step represent the most commercially viable resolution pathway.

### 3.1 Physical vapour deposition (PVD) methods

Physical vapour deposition (PVD) methods, particularly magnetron sputtering and plasma electrolytic oxidation (PEO/MAO), provide the highest adhesion strength through *in situ* oxide bond formation on valve metal substrates (Ti, Al, Mg), but are restricted to planar or electrically conductive substrates and low-throughput batch processes; Bi et al. exploited MAO as a structural base layer in their trifunctional Mg alloy implant coating (Bi et al., 2026), while Lv et al. demonstrated that PEO in CuO-containing electrolytes produces dense, corrosion-resistant ceramic coatings (Lv et al., 2024), and Husak et al. showed that CuO incorporation into PEO on pure Mg simultaneously reduces magnesium ion release and promotes bioactive hydroxyapatite crystallisation in simulated body fluid (Husak et al., 2024).

### 3.2 Wet chemical methods

Wet chemical methods (dip coating, spray coating) are the most industrially accessible and substrate-universal: Rai et al. demonstrated an end-to-end gram-scale synthesis to dip-coat workflow on filter paper (Rai, 2023); Foffa et al. spray-coated a PU/CuNP composite onto face mask substrates, achieving >99% SARS-CoV-2 inactivation within 30 min (Foffa et al., 2022).

### 3.3 The textile-specific pad-dry-cure (PDC) process

The textile-specific pad-dry-cure (PDC) process, the industrial standard for functional textile finishes, operating at 30–100 m/min, is highly scalable but produces surface-localised NP deposits that detach under laundering unless augmented by covalent anchoring pre-treatments; Hu et al. solved this with a polydopamine (PDA) interlayer on PET, achieving 40-cycle durability (GB/T 12490-2014) (Hu et al., 2022). Layer-by-layer (LbL) deposition provides unmatched nanoscale architectural control and biocompatibility, demonstrated by poly(amino acid) multilayers with embedded Ag/CuO NPs achieving both antibacterial activity and mammalian cell compatibility, but remains primarily a research tool due to multi-step processing constraints (Kruk et al., 2019; Milisav et al., 2025).

### 3.4 Polymer nanocomposite casting

Polymer nanocomposite casting, dispersing CuNMs in epoxy (El Saeed et al., 2016; Kang et al., 2025), PDMS (Tavakoli et al., 2019), PU (Foffa et al., 2022), or biopolymer (Dardari et al., 2025) matrices, offers the widest substrate scope and is directly compatible with existing industrial coating lines, making it the most commercially promising fabrication class. Polydopamine-assisted electroless deposition of Cu NPs has further expanded substrate options to include reverse osmosis membranes (Liu et al., 2022) and wood surfaces (Duan et al., 2020), demonstrating the universality of catechol-based anchoring chemistry.

As summarised in Table 2, the selection of a coating fabrication method fundamentally dictates the operational boundaries of the resulting antimicrobial system across adhesion, substrate scope, wash durability, scalability, and transparency potential. A comparative evaluation of these techniques reveals a persistent structural trade-off: methods offering the highest industrial scalability, such as dip/spray coating, textile pad-dry-cure, and polymer nanocomposite casting, intrinsically suffer from lower baseline adhesion and wash durability compared to highly adherent but poorly scalable vacuum-based or electrolytic techniques like magnetron sputtering and PEO/MAO. Consequently, the most promising trajectory for resolving this dichotomy lies in hybrid architectures. By coupling a high-adhesion base layer (such as PEO, MAO, or a universal polydopamine interlayer) with a highly scalable functional layer application, researchers can effectively bridge the gap between rigorous durability requirements and commercial manufacturing viability.

**TABLE 2:** Comparative evaluation of CuNM coating fabrication methods across key performance criteria: adhesion strength, substrate scope, wash durability, scalability, and optical transparency potential.

Method	Adhesion	Substrate scope	Wash durability	Scalability	Transparency potential	Key references
Magnetron sputtering	Very high	Rigid planar	Moderate	Low (batch/vacuum)	Good (<50 nm film)	Gutnik et al. (2026), Khalifa et al. (2025)
PEO/MAO	Exceptional	Valve metals only	Very high	Low (batch)	None (dense oxide)	Bi et al. (2026), Husak et al. (2024), Lv et al. (2024)
Dip/spray coating	Moderate	Universal	Moderate (5–20 cycles)	High (roll-to-roll)	Good (thin layer)	Foffa et al. (2022), Rai (2023)
Pad-dry-cure (textile)	Moderate	Textiles	Low–moderate (10–20)	Very high	N/A	Hu et al. (2022), Karypidis et al. (2023), Singh et al. (2023)
*In situ* synthesis	High (fibre integration)	Textiles, porous	High (20–40+ cycles)	Moderate	N/A	Chen et al. (2025a), Ran et al. (2021)
LbL assembly	High (electrostatic)	Charged surfaces	High	Low–moderate	Very good	Kruk et al. (2019), Milisav et al. (2025)
Polymer nanocomposite	High (entrapment)	Universal	High (matrix-dep.)	High	Moderate	El Saeed et al. (2016), Kang et al. (2025), Tavakoli et al. (2019)

Qualitative ratings are derived from corpus synthesis. Hybrid architectures coupling a high-adhesion base layer (PEO/MAO or polydopamine interlayer) with a scalable functional deposition step are identified as the most commercially promising pathway for resolving the adhesion–scalability trade-off.

Surface pre-treatment is the underappreciated determinant of coating adhesion and durability, yet is omitted from the reporting of the majority of corpus fabrication studies. For metal substrates, combined degreasing, acid etch, controlled abrasion (surface roughness R_z_ 2–5 µm), and oxidative plasma treatment increase surface energy from ~30 mJ/m^2^ (contaminated steel) to >70 mJ/m^2^ (hydroxyl-terminated), directly translating to improved adhesive bond strength measurable by pull-off adhesion testing (ASTM D4541).

For textile substrates, desizing and scouring remove finishing agents that reduce fibre surface energy; alkali or permanganate pre-oxidation introduces hydroxyl and carboxyl groups that serve as nucleation sites for *in situ* CuNM synthesis or as anchoring sites for pre-formed NPs (Agrawal et al., 2020). For polymeric hard surfaces, UV-ozone, O_2_ plasma, or corona treatment increases polarity and wettability, directly correlating with improved coating adhesion under subsequent mechanical testing.

Despite their central importance, pre-treatment protocols are reported in full detail in fewer than 30% of corpus fabrication studies, a significant reproducibility barrier (Singh et al., 2023). Recent smart textile approaches, including sensor-integrated bactericidal systems that provide *in-situ* visual feedback on antimicrobial efficacy (Ferrer-Vilanova et al., 2025), point toward the next fabrication generation where function and performance monitoring are co-integrated. The proposed MRS (Section 9) explicitly requires surface pre-treatment reporting as one of its eight mandated parameters; without this information, adhesion performance data across different laboratories cannot be interpreted or reproduced (Glazkova et al., 2022; Hu et al., 2022; Karypidis et al., 2023).

## 4 Ion release mechanisms and controlled release strategies

Antimicrobial efficacy is ultimately mediated by Cu^2+^ delivery at concentrations above organism MIC, making the ion release profile, time-dependent rate, duration, and spatial pattern of Cu^2+^ delivery, the mechanistic link between coating structure and biological activity (Figures 3, 4). In passive dissolution systems, Cu oxide solubility increases ~10-fold per pH unit below 7; at the sweat pH range (4.0–5.5), release rates are 2 orders of magnitude higher than at neutral pH (Tavakoli et al., 2019). Temperature effects (Arrhenius kinetics), NP size (surface area scales inversely with diameter, following Kelvin equation), and polymer matrix diffusion resistance (described by the Higuchi square-root-of-time model for matrix-embedded systems) together determine the release profile from any given coating architecture (El Saeed et al., 2016; Gangadoo et al., 2020).

**FIGURE 4:**
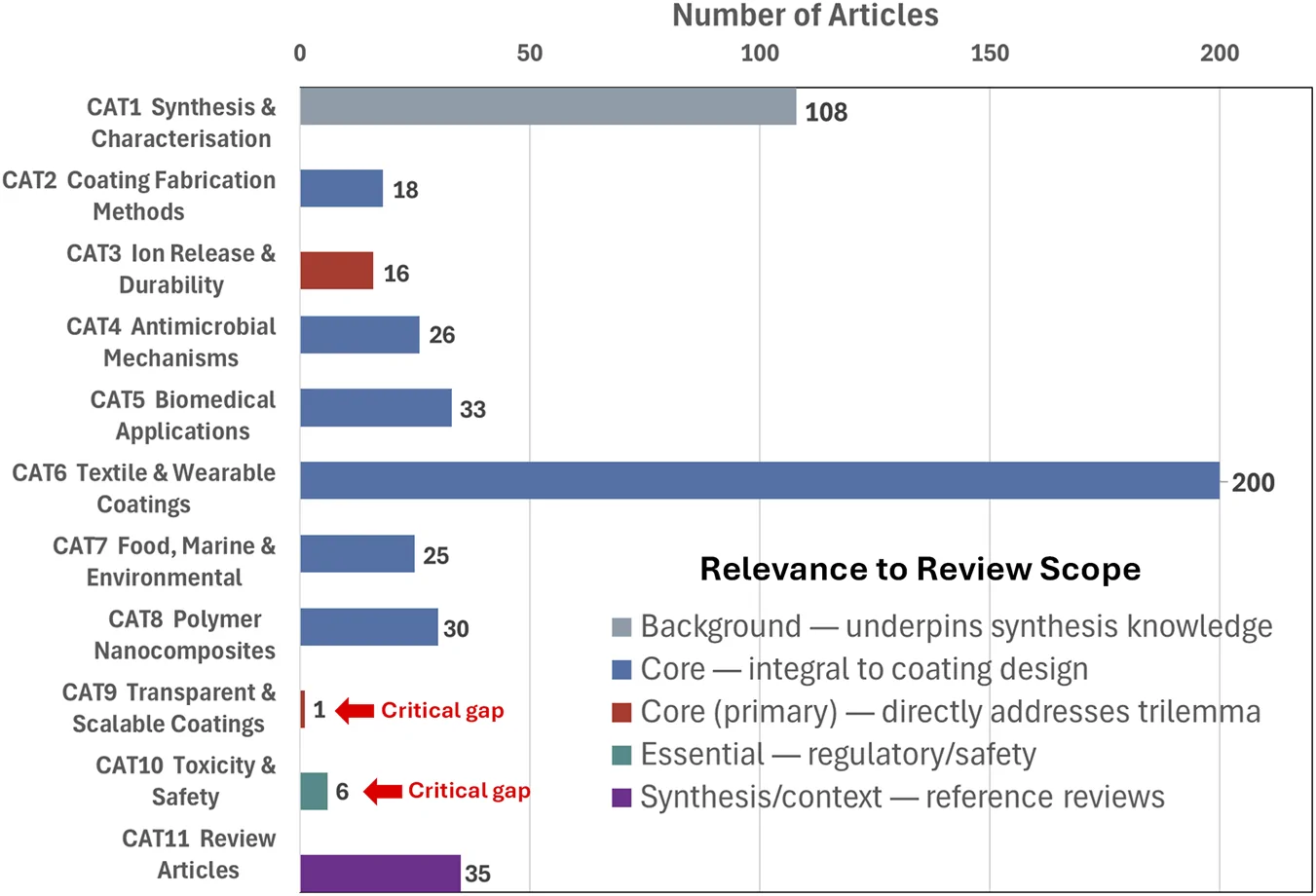
Controlled ion release strategies in CuNM coating systems. Passive dissolution is governed by pH (Cu oxide solubility increases ~10-fold per unit below pH 7), NP size (surface area ∝ 1/diameter), and polymer matrix diffusion resistance (Higuchi square-root-of-time kinetics). Stimulus-responsive architectures decouple release from constant passive dissolution: pH-responsive systems activate selectively in the acidic infection microenvironment (pH 5.5–6.5); NIR-triggered CuS NPs provide on-demand photothermal release; dual-stimulus Cu-MOF frameworks respond to combined pH decrease and elevated humidity, enabling targeted Cu^2+^ delivery with reduced off-target exposure.

Quantitative benchmarking of ion release rates across corpus studies reveals striking variability driven by both genuine system differences and reporting inconsistencies. For polymer matrix-embedded CuO NPs, reported Cu^2+^ release rates span from 0.02 μg/cm^2^/day in dense epoxy matrices at 2 wt% loading (El Saeed et al., 2016) to >5 μg/cm^2^/day in porous chitosan films at equivalent Cu content, a 250-fold range reflecting primarily matrix diffusion resistance. For CuNP-textile systems without polymer matrix entrapment, burst release in the first wash cycle can account for 40%–70% of total Cu inventory (Singh et al., 2023), rendering long-term efficacy unsustainable. The minimum effective Cu^2+^ surface flux for Gram-positive bacterial inhibition in standardised contact-kill models is approximately 0.05–0.1 μg/cm^2^/day for *S. aureus* ATCC 6538 (Gangadoo et al., 2020; Tavakoli et al., 2019); biofilm inhibition requires 2–5-fold higher fluxes due to the extracellular polysaccharide diffusion barrier. For Gram-negative *Escherichia coli*, effective fluxes are 3–5-fold higher still. These thresholds define the engineering design space: Cu^2+^ flux must be maintained above the organism-specific minimum effective concentration for the target service lifetime while remaining below cytotoxicity thresholds, onset at approximately 50–100 mg/L Cu^2+^ for human cell lines, equivalent to 10–20 μg/cm^2^/day at typical contact area assumptions. The narrow therapeutic window is most constraining in biomedical implant applications where intimate tissue contact makes over-release directly harmful, and where the pH-responsive and stimulus-triggered systems described below were specifically designed to address this challenge (Bi et al., 2026; Bose et al., 2025; Tavakoli et al., 2019).

Stimulus-responsive systems decouple Cu^2+^ release from constant passive dissolution, activating delivery only when antimicrobial activity is needed. The most compelling pH-responsive system is Bi et al.’s MAO/PCL@GC coating on AZ31B Mg alloy implants, in which Cu_2_O NPs on graphene oxide (GC NPs) embedded in polycaprolactone release Cu^2+^ at accelerated rates specifically in the acidic infection microenvironment (pH 5.5–6.5), providing autonomous infection-responsive defence without continuous Cu^2+^ exposure in sterile healing phases (Bi et al., 2026). Men et al. extended dual-stimulus control to food packaging with α-lipoic acid (α-LA)-functionalised Cu-MOF (LA@Cu-MOF) that responds to both pH decrease and elevated headspace humidity, demonstrating 6-day fresh-produce preservation compared to unfunctionalised controls (Men et al., 2025). NIR photothermal release is most efficiently implemented with CuS NPs, whose strong NIR absorption (900–1100 nm) converts photon energy to local hyperthermia (>50 °C), simultaneously killing bacteria through thermal denaturing, accelerating Cu^2+^ dissolution, and generating ROS through photothermal-enhanced Fenton chemistry; Bose et al. (2025) demonstrated NIR-II (1064 nm) activation on nanoporous Ti implant surfaces, while Sun et al. (2024), He et al. (2024), and Chen et al. (2023) have documented dual antibacterial-wound healing functions in CuS-based wound dressing platforms; Yan et al. (2024) and Qiao et al. (2023) extended NIR photothermal therapeutic functionality to mesoporous PDA/CuS and hollow CuS architectures respectively, demonstrating tuneable burst-release profiles through cavity geometry design.

## 5 Durability and long-term efficacy

Durability failure occurs through four principal mechanisms: (i) physical NP detachment (dominant for surface-localised coatings without covalent anchoring) (Hu et al., 2022; Karypidis et al., 2023), (ii) chemical depletion of the Cu^2+^ reservoir, (iii) oxidation state drift (Cu^0^/Cu_2_O → CuO under ambient conditions, reducing ion release rate and worsening optical properties, a process characterised by time-resolved XPS showing progressive surface hydroxylation and Cu(OH)_2_/malachite phase growth over months of atmospheric exposure (Batenkova et al., 2026), and (iv) polymer matrix degradation by UV, bleach, solvents, or enzymatic attack (Glazkova et al., 2022; Khalifa et al., 2025). The most reliable durability engineering strategy in the corpus is catechol-based surface anchoring (Table 3): polydopamine (PDA) forms conformal, multi-bond adhesion layers on virtually any substrate through coordinate bonding (catecholate-Cu^2+^), Michael addition, π-π stacking, and hydrogen bonding. Hu et al. applied a PDA interlayer on PET to anchor graphene oxide/Cu_2_O (GO/Cu_2_O) composite nanoparticles, establishing a corpus benchmark of 90% efficacy retention against *S. aureus* and 88% against *E. coli* after 40 laundering cycles (GB/T 12490-2014 standard) (Hu et al., 2022). Ran et al. complemented this by engineering Cu_2_O@CAT-TEPA core-shell particles in which the catechol-terminated polyamine shell simultaneously stabilises the Cu_2_O core against further oxidation to the less active CuO (chemical stability) and provides surface coordination sites for substrate anchoring (mechanical stability), conferring improved chemical and water-immersion stability, with CuO formation fully suppressed after 20 days of aqueous immersion, relative to unmodified Cu_2_O NPs (Ran et al., 2021). Chen et al. advanced waterborne polyurethane (WPU)-capped Cu_x_O NPs (average diameter 3.55 nm) on cotton as the corpus’s highest-reported wash durability benchmark: >99.98% efficacy against both *S. aureus* and *E. coli* maintained after 50 laundering cycles (GB/T 12490-2014), attributed to WPU’s amphiphilic self-assembly forming a strongly adhesive film with metal-N bonding to the Cu particle surface (Chen B. et al., 2025). Polymer matrix physical entrapment provides an independent retention mechanism: NPs geometrically constrained within crosslinked polymer meshes cannot diffuse out under operational stresses, provided that NP diameter exceeds the matrix mesh size (Gangadoo et al., 2020; Glazkova et al., 2022; Tavakoli et al., 2019).

**TABLE 3:** Wash durability benchmarks from representative textile CuNM coating studies, organised by CuNM form, anchoring strategy, wash test standard, cycle count, and retained antibacterial efficacy.

Cu form	Anchoring strategy	Standard	Cycles	Efficacy retained	Study
Cu_2_O on GO	PDA interlayer on PET	GB/T 12490-2014	40	90% *S. aureus*; 88% *E. coli*	Hu et al. (2022)
Cu_2_O@CAT-TEPA	Core-shell + catechol anchor	Water immersion (20 days)	Not specified	CuO formation suppressed; oxidation/immersion stability (not laundering)	Ran et al. (2021)
Cu NPs composite	LbL/polymer matrix	ISO/AATCC	15–30	Variable by anchor type	Karypidis et al. (2023)
CuO NPs	Conventional PDC binder	Various	10–20	Significant loss	Singh et al. (2023)
Ag NPs on Cu (ion-exchange)	Silane + ion-exchange reduction	Custom protocol	7 days	Active (Ag-mediated)	Gangadoo et al. (2020)
WPU-capped CuxO NPs	Amphiphilic WPU film (*in situ* reduction)	GB/T 12490-2014	50	>99.98% *S. aureus* and *E. coli*	Chen et al. (2025a)

AATCC 61, ISO 6330, and GB/T 12490-2014 use different temperatures, detergents, and mechanical actions; no validated inter-standard conversion factor is available, precluding direct cross-study comparison. Standardised cleaning-durability data for CuNM hard surface coatings are absent from this corpus (Supplementary Dataset S1, Sheet 5), representing a critical near-term evidence gap.

Wash durability testing standards vary substantially across the corpus, preventing direct cross-study comparison of reported cycle counts. AATCC Test Method 61 (USA) uses sodium carbonate detergent at 49 °C; ISO 6330 (International) specifies ECE reference detergent at 40 °C or 60 °C, producing different mechanical shear, thermal, and chemical degradation profiles with no validated conversion factor reported in any corpus study. Accelerated wash tests in AATCC simulate home laundering through higher temperature and mechanical action, but the relationship to ISO 6330 wash cycles differs substantially by substrate type and NP anchor chemistry. For hard surface coatings, the standardisation deficit is even more severe: studies variously report Taber abrasion (ASTM D4060), cross-hatch adhesion (ASTM D3359), sequential ethanol wiping, or alkaline solution immersion with inconsistently reported contact times and chemical concentrations. UV accelerated ageing (ASTM G154), salt spray (ASTM B117), and pencil hardness (ASTM D3363) are standard requirements in industrial hard surface coating specifications but are absent from virtually all corpus CuNM coating studies. Without a common test platform, a coating passing its own authors’ durability criteria may fail entirely under an industrial qualification protocol, creating a persistent valley of death between academic proof-of-concept and commercial adoption A separate question is whether the existing standards would be adequate if they were used. For antimicrobial efficacy they largely are: ISO 22196 and JIS Z 2801 for hard non-porous surfaces, ISO 20743, AATCC 100 and JIS L 1902 for textiles, and ISO 21702 for antiviral activity are established and quantitative. The mechanical durability standards cited above are likewise fit for their stated purpose, but none carries an antimicrobial endpoint, and the laundering standards define a method without defining either a pass criterion or a conversion between AATCC 61, ISO 6330 and GB/T 12490-2014. Two protocols already couple ageing to efficacy in the manner this field requires. ASTM E3162-18 launders treated textiles under accelerated conditions expressly so that standard antibacterial assays can be run on the aged material, and recognises 5, 25 and 50 washes as durability tiers. EPA Protocol 01-1A, with method MB-41, applies wet and dry wear cycles with re-inoculation to hard non-porous surfaces before a final efficacy test (Environmental Protection Agency, 2015). Neither appears in any corpus study. The deficiency is therefore not an absence of standards but their non-adoption, together with three extensions that CuNM systems specifically require and that no current standard provides: confirmation of oxidation state before and after ageing, since the Cu^0^ to Cu_2_O to CuO progression alters both ion release and colour; quantification of residual Cu inventory rather than pass or fail efficacy retention, without which remaining service life cannot be predicted; and re-measurement of transmittance and haze after abrasion, the property most decisive for transparent applications. Extending ASTM E3162-18 and EPA Protocol 01-1A to CuNM coatings would be more tractable than writing new standards from first principles (Karypidis et al., 2023; Khalifa et al., 2025; Singh et al., 2023).

For hard surface coatings, the application class with the highest commercial value for transparent antimicrobial applications, durability data are nearly absent from the corpus. Only 22 of the 194 non-textile articles with retrievable full text (11.3%) report any abrasion, scratch or cleaning-cycle durability testing; none applies a standardised disinfectant-wipe protocol to a CuNM coating on a transparent hard substrate, and none reports efficacy retention beyond 100 sequential cleaning cycles (Supplementary Dataset S1, Sheet 5). Hard surface adhesion challenges differ fundamentally from textiles: thin-film coatings must withstand combined mechanical shear (10–50 N fingertip load), chemical exposure (70% isopropanol, quaternary ammonium solutions, sodium hypochlorite), and UV photooxidation, all within a transparent geometry where maximum acceptable film thickness is ~100–200 nm before visible optical artefacts appear. Sol-gel derived silica hybrid matrices are the most industrially mature hard surface coating chemistry, achieving pencil hardness up to 6H and >500 Taber abrasion cycles in commercial architectural glass coatings without CuNM functionality. Integration of CuNMs into sol-gel silica matrices at <0.1 wt% loading has been demonstrated at proof-of-concept level (Glazkova et al., 2022; Luo et al., 2025), but comprehensive durability characterisation under standardised industrial test protocols remains unreported and represents a critical near-term research priority, arguably the single most commercially valuable experiment the transparent CuNM coating field could perform (Glazkova et al., 2022; Kang et al., 2025; Luo et al., 2025; Pellosi et al., 2025).

## 6 Optical transparency: the most commercially decisive property

Optical transparency is treated as a design objective by only eight of 498 corpus articles (1.6%), of which just three report a quantitative visible transmittance value (Supplementary Dataset S1, Sheet 4), yet it determines commercial viability for the largest application markets: touchscreens, glass partitions, food packaging windows and medical device housings. The ≥80% criterion adopted in this review is the minimum below which a coating is commercially non-viable across all transparency-critical applications; the most demanding of them, display cover glass and architectural glazing, require ≥90% at 550 nm and are treated separately in Table 4. The physical origins of coloration differ by Cu species: Cu0 NPs exhibit LSPR at 570–590 nm (orange-red, scales as NP volume, not eliminable by size reduction alone) (Khalifa et al., 2025; Luo et al., 2025; Pellosi et al., 2025); Cu_2_O NPs absorb at ~571 nm (direct bandgap) but absorption diminishes for particles <15 nm via quantum confinement, making Cu_2_O quantum dots (3–5 nm) the most promising oxide species for transparent systems (Luo et al., 2025; Ran et al., 2021); CuO NPs absorb broadly across the entire visible range through d-d and charge-transfer transitions, intrinsically opaque regardless of particle size (Glazkova et al., 2022; Tavakoli et al., 2019); CuS NPs absorb primarily in the NIR (900–1100 nm), producing only a faint tint at loadings <0.1 wt%, making them the most optically compatible CuNM form (Bose et al., 2025; Sun et al., 2024); Cu-MOF frameworks achieve >80% transmittance below ~0.5 wt% loading with acceptable teal-blue tint (Hubab et al., 2025; Men et al., 2025).

**TABLE 4:** Application-sector requirements matrix for antimicrobial CuNM coatings across six deployment sectors: biomedical implants, wound dressings, healthcare textiles, food packaging, high-touch transparent surfaces, and marine antifouling.

Sector	Key substrate(s)	Efficacy target	Optical req.	Durability req.	Regulatory path	TRL	Key references
Biomedical implants	Ti, SS, PEEK	≥4-log MRSA	None (opaque)	Implant lifetime (years)	FDA 510(k); ISO 10993	4–6	Bose et al. (2025), Gao et al. (2025), Han et al. (2023), Tavakoli et al. (2019), Zhao et al. (2026)
Wound dressings	Hydrogel, PU foam	≥3-log + biofilm	Not required	1–7 days (single use)	FDA Class II/III	5–7	Bi et al. (2026), Chen et al. (2023), He et al. (2024), Sun et al. (2024)
Healthcare textiles	Cotton, PET, nylon	≥2-log; ISO 20743	Not required	≥50 wash cycles	AATCC self-regulated	6–7	Ferrer-Vilanova et al. (2025), Foffa et al. (2022), Glazkova et al. (2022), Karypidis et al. (2023), Kumeleh et al. (2025), Singh et al. (2023)
Food packaging	PET, PP, biopolymer	Shelf-life ext.; <5 mg/kg migration	High (windows)	Shelf-life duration	EU 10/2011; EU BPR	4–5	Gopinath et al. (2024), Mesgari et al. (2022), Reijnders (2023)
High-touch surfaces	Glass, PC, acrylics	≥2-log; EPA 24 h	>90% transmittance	>3 months	EPA FIFRA	3–4	El Saeed et al. (2016), Gangadoo et al. (2020), Glazkova et al. (2022), Pellosi et al. (2025)
Marine antifouling	Steel, FRP	Biofouling prevention	None	≥5 years (hull)	EU BPR; IMO	5–6	Thai et al. (2023)

For each sector, quantitative efficacy targets, optical transmittance requirements, durability standards, regulatory pathway, and Technology Readiness Level (TRL) are specified. High-touch transparent surfaces (TRL 3–4) impose the most demanding combined requirements (≥90% T at 550 nm and ≥2-log EPA 24 h kill) and represent the field’s most commercially critical, least-addressed development gap.

Quantitative transmittance benchmarks from the three corpus studies reporting a numerical visible transmittance value, together with the qualitative descriptions given by the remaining transparency-oriented studies, define the current performance frontier by CuNM species (Supplementary Dataset S1, Sheet 4). For Cu^0^ NP films, even sub-monolayer deposition at <0.01 wt% loading reduces transmittance by 15%–30% at 570 nm, the Cu^0^ LSPR maximum and therefore the worst case across the visible range, due to LSPR extinction; maximum reported transmittance for Cu^0^ NP-functional coatings with verified bactericidal activity is approximately 75% at 550 nm, below the commercial threshold, with orange-yellow coloration clearly perceptible to the human eye (CIE ΔE > 3) (Khalifa et al., 2025; Pellosi et al., 2025). For Cu_2_O NPs at conventional sizes (20–100 nm), achieving 80% transmittance requires loading <0.05 wt%, which delivers insufficient Cu inventory for sustained bactericidal activity beyond a single wash cycle. Luo et al. partially resolved this by assembling Cu_2_O clusters on high-surface-area scaffold fibres, achieving local Cu_2_O concentrations sufficient for activity while maintaining area-averaged transmittance through sub-wavelength feature geometry, an architectural principle applicable to other substrate types (Luo et al., 2025). CuS NPs at <0.1 wt% loading achieve >85% transmittance at 550 nm with confirmed NIR-activated photothermal antibacterial activity, the only corpus entry in the commercially viable upper-right quadrant of a transparency-vs-efficacy performance map (Bose et al., 2025; Sun et al., 2024). Cu-MOF composites at 0.3–0.5 wt% loading show 82%–87% transmittance with mild teal tint, relying on synergistic secondary antimicrobials to compensate for reduced Cu^2+^ flux at sub-coloration Cu concentrations. Practical film uniformity presents an additional challenge: >5 nm thickness variation across 10 cm^2^ creates visible optical inhomogeneity, restricting viable deposition processes to vapour-phase or spin-coat methods that are incompatible with cost-effective roll-to-roll mass production (Hubab et al., 2025; Men et al., 2025).

The transparency–durability–efficacy trilemma can be formulated quantitatively. Efficacy (E) scales with surface Cu^2+^ concentration relative to organism MIC, requiring higher Cu loading (L) (Figure 5). Transparency (T) decreases with L as T = f(L, d, species, matrix); switching to less-absorbing species (CuS > Cu_2_O > Cu0 > CuO) or reducing NP size d partially decouples T from L. Durability (D), in the sense of mechanical adhesion, is largely independent of L; but efficacy duration (D′) scales linearly with L for constant release rate, lower loading coatings are antimicrobially exhausted faster. Thus, E and T are in direct tension at fixed species and size, while D' (not D) is also in tension with T. The only routes to resolving the trilemma are: switching to lower-absorption Cu species (CuS, Cu_2_O QDs, Cu-MOF); reducing NP size to shift the absorption-to-activity ratio; adopting regenerable architectures that maintain efficacy without large Cu reservoirs; or combining sub-threshold CuNM loading with secondary antimicrobial agents to maintain efficacy below the coloration threshold (Khalifa et al., 2025; Pellosi et al., 2025).

**FIGURE 5:**
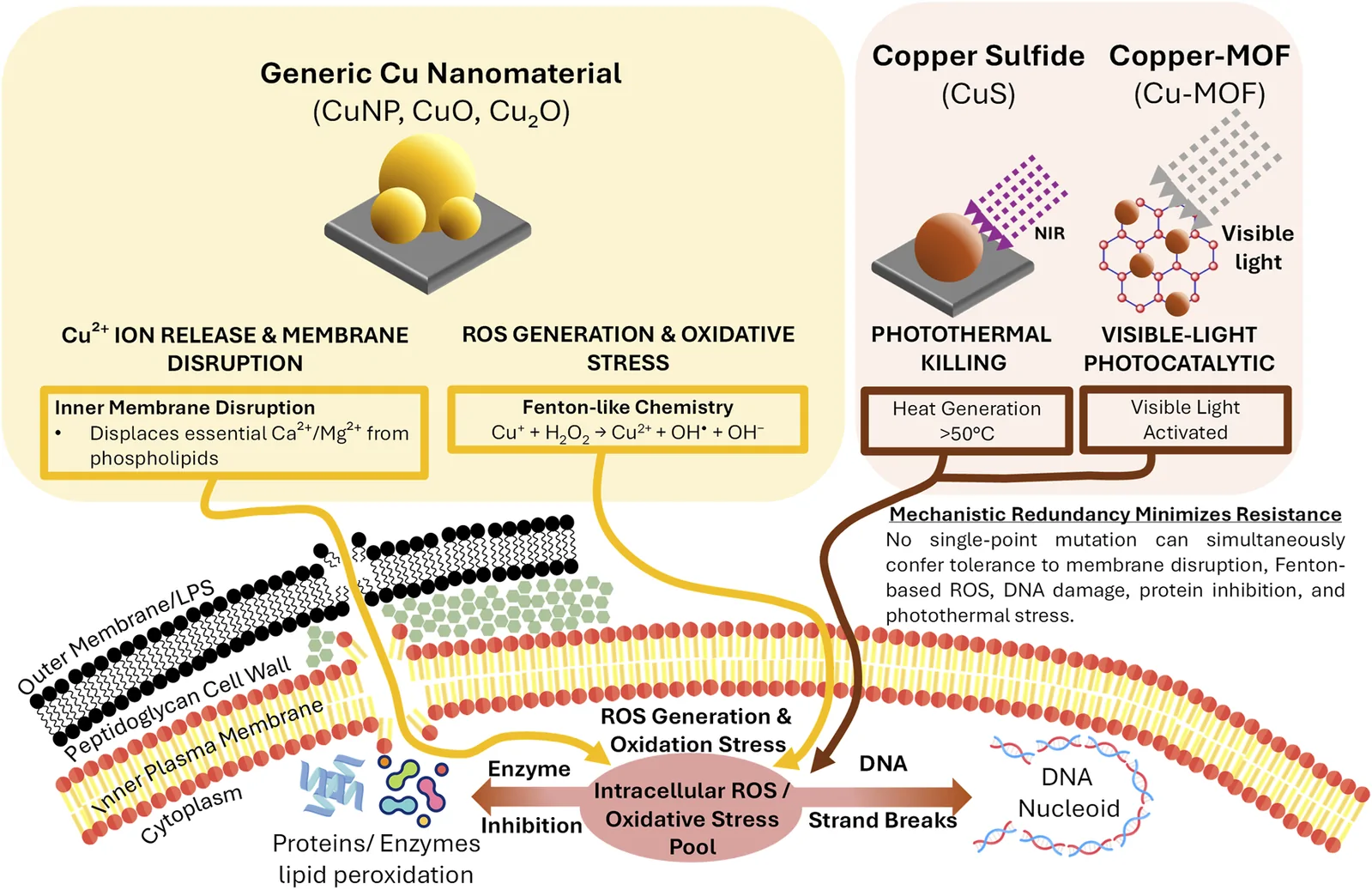
Transparency–durability–efficacy trilemma in copper nanomaterial (CuNM) antimicrobial coatings. Antibacterial efficacy (E, log_10_ CFU reduction) is plotted against optical transmittance (T, % at 550 nm); marker area encodes wash-cycle durability (D). Dashed lines at T = 80% and E = 2-log demarcate the commercially viable zone (upper right). CuS NPs are the only CuNM class with demonstrated entries in this zone; Cu^0^ and CuO NPs are confined to the high-efficacy, low-transparency region. Arrows indicate resolution pathways: lower-absorption Cu species (CuS, Cu_2_O QDs, Cu-MOF), smaller NP size, and regenerable architectures.

## 7 Application-specific performance assessment

Performance requirements differ substantially across application sectors, and a coating architecture optimised for one context will typically fail the requirements of another. Table 4 provides the quantitative requirements matrix. CuNM coating performance is benchmarked against these sector-specific targets.

### 7.1 Biomedical applications

Implant-associated infection (IAI) affects 2%–5% of orthopaedic implants and up to 8% of vascular access devices, imposing a USD 10 billion annual burden in the US (Bose et al., 2025; Li et al., 2021). CuNM coatings address IAI by delivering bactericidal Cu^2+^ directly at the surface where biofilm initiation occurs. Li et al. demonstrated on PCL electrospun membranes that a mussel-inspired hyperbranched polyglycerol (MI-hPG) coating simultaneously reduces bacterial adhesion (antifouling), releases bactericidal Cu^2+^, and promotes osteoblastic differentiation, addressing both IAI and osseointegration failure with a single architecture (Li et al., 2021). Tavakoli et al. characterised the therapeutic window for CuO NP-PDMS-SiO_2_ on 316L SS: below 2 wt% CuO, corrosion resistance improves and biocompatibility is maintained; above 1 wt%, cytotoxicity to MG63 osteoblast-like cells becomes significant (Tavakoli et al., 2019). The wound care sector represents the highest TRL (5–7) among all application areas, with *in vivo* efficacy validation in rodent models provided by Sun et al. (2024), He et al. (2024), Chen et al. (2023), and Bi et al. (2026), each demonstrating multi-functional CuNM platforms addressing infection, inflammation, and tissue remodelling simultaneously. On implant surfaces, Osman et al. (2025) and Zhao et al. (2026) provide dual antibacterial/osteogenic strategies on titanium that combine Cu^2+^ bactericidal activity with surface topography for osseointegration promotion; Han et al. comprehensively review stimuli-responsive nanotechnologies across orthopedic Ti implant platforms (Han et al., 2023); and Gao et al. demonstrate NIR photothermal Cu NPs achieving >99% bacteria kill on Ti implant surfaces under 808 nm irradiation (Gao et al., 2025); Bose et al. demonstrated an earlier NIR-responsive system combining Cu_2_O@CuFeS_2_ composite with chitosan-hydroxyapatite matrix on titanium, achieving photothermal conversion efficiency of ~39% under 808 nm NIR irradiation for implant antibacterial applications (Bose et al., 2023). Zhao et al. further demonstrated that Ti-Cu alloy coatings promote osseointegration by activating macrophage-mediated immunomodulation, accelerating the M1-to-M2 macrophage phenotype transition and creating a pro-healing microenvironment that is complementary to direct bactericidal Cu^2+^ action on Ti implants (Zhao et al., 2023).

### 7.2 Textile, wearable, and PPE applications

Healthcare textiles constitute the corpus’s largest application cluster (200 articles, CAT6). The heightened pandemic-era demand for antimicrobial PPE generated the corpus’s most direct virucidal evidence: Foffa et al. achieved >99% SARS-CoV-2 inactivation within 30 min on spray-applied PU/CuNP face mask substrates, mechanistically distinct from bacterial killing through Cu^2+^ oxidative attack on viral envelope lipids and spike protein (Foffa et al., 2022); Giedraitienė et al. systematically demonstrated that CuO NPs achieve equal or superior broad-spectrum activity against both Gram-positive (*S. aureus*) and Gram-negative (*K. pneumoniae*) bacteria relative to AgNPs on comparable substrates, confirming CuO as a robust multi-pathogen PPE surface coating candidate (Giedraitienė et al., 2022). Singh et al. and Karypidis et al. independently identified wash durability as the primary commercial challenge, with most NP-based textile finishes losing significant efficacy within 10–20 wash cycles without specialised anchoring chemistry; CuNP-based textiles consistently outperform QAC equivalents on breadth of spectrum and wash durability, while QAC textiles retain cost and regulatory simplicity advantages (Karypidis et al., 2023; Singh et al., 2023). Recent industrially-focused advances include eco-friendly Cu NP synthesis routes compatible with textile finishing (Kumeleh et al., 2026) and smart indicator textiles providing real-time visual confirmation of antimicrobial status (Ferrer-Vilanova et al., 2025), both addressing commercial adoption barriers (Chen B. et al., 2025); Chen et al. introduced lignin-copper composite nanoparticles as a sustainable bio-based approach, leveraging lignin’s natural hierarchical structure as both Cu-reduction scaffold and structural matrix, achieving ≥3-log *S. aureus* and *E. coli* reductions with enhanced respiratory protection metrics in mask-format substrates (Chen M. et al., 2025).

A significant methodological concern in the textile antimicrobial literature is the inappropriate application of zone-of-inhibition (ZOI) testing to CuNM systems. ZOI halo tests detect volatile or freely diffusible biocidal agents, appropriate for AgNP systems where Ag^+^ diffuses readily through agar, but are mechanistically unsuitable for CuNM coatings whose Cu^2+^ activity is primarily contact-dependent. Of the 109 textile studies for which full text was retrievable, 40 (36.7%) rely on ZOI methods without reporting any quantitative contact test, and only 23 (21.1%) report a standardised contact assay (Supplementary Dataset S1, Sheet 6); ZOI-only reporting generates false-negative results and underestimates true contact-kill performance. The correct benchmarks are quantitative contact tests: ISO 20743, AATCC 100, and JIS L 1902, each reporting log-reduction values from direct surface contact over defined time periods. Studies using ISO 20743 or AATCC 100 consistently report 2–4 log reductions against *S. aureus* and *E. coli* for adequately loaded CuNM textile finishes with appropriate anchoring chemistry. The direct comparison by Karypidis et al. (2023) across multiple anchoring methods demonstrated that LbL-assembled coatings outperformed PDC-only and spray-only methods on both wash durability and quantitative contact kill, but at 3–4-fold higher processing cost per linear metre, a trade-off not quantified through explicit cost-efficiency metrics in any corpus publication, leaving technology selection decisions without rigorous economic evidence (Foffa et al., 2022; Hu et al., 2022).

### 7.3 Food packaging

European Union Regulation 10/2011 imposes a specific Cu migration limit of 5 mg/kg food, creating a hard ceiling on Cu loading in food contact coatings that simultaneously constrains ion release rate. Men et al. navigated this with dual pH/humidity-responsive LA@Cu-MOF films that exploit α-lipoic acid’s independent antioxidant and antimicrobial activity, permitting below-SML Cu loading while maintaining 6-day fresh-produce preservation compared to unfunctionalised controls (Men et al., 2025). Mesgari et al. reviewed evidence that CuO NPs in biopolymer matrices (chitosan, starch, PLA) extend fresh produce shelf life by 2–5 days (Mesgari et al., 2022). Dardari et al. added thermal buffering via phase-change material (PEG-grafted cellulose nanocrystals/Cu NPs) in carrageenan/PVA, providing both antimicrobial function and cold-chain temperature regulation (Dardari et al., 2025). Gopinath et al. provide the most comprehensive overview of CuO nanofiller integration in food packaging systems, cataloguing shelf-life extensions of 4–21 days across diverse commodity types and documenting Cu migration levels from 0.024 to 0.65 mg/kg across different food simulants, below the 5 mg/kg EU Regulation 10/2011 threshold in optimised formulations (Gopinath et al., 2024).

Active packaging based on CuNM antimicrobial films must navigate the dual requirement of maintaining food safety, Cu migration strictly below 5 mg/kg under EU Regulation 10/2011, while achieving demonstrable shelf-life extension that justifies premium packaging costs. Matrix design directly manages this trade-off: Cu-MOF in compact polymer matrices (PLA, PET) releases Cu^2+^ primarily through pore diffusion at rates well below the specific migration limit, while supplementary antimicrobial activity from α-lipoic acid (α-LA) (Men et al., 2025) or biopolymer matrix components compensates for reduced Cu^2+^ flux at sub-SML Cu loadings. The commercial opportunity is substantial: the global active and intelligent food packaging market exceeded USD 18 billion in 2024 and is projected at 6%–8% CAGR through 2030, with antimicrobial films as the fastest-growing segment driven by post-pandemic food safety awareness and retailer sustainability commitments. CuNM packaging systems are well-positioned relative to Ag-based alternatives on cost grounds (approximately 150-fold feedstock cost advantage), but regulatory approval timelines under EU 10/2011 and EFSA safety assessment requirements are the primary time-to-market barrier. A focused development programme targeting a specific high-value commodity (strawberries, leafy greens, or fresh-cut produce) with a single well-characterised CuNM formulation would represent the most efficient regulatory submission pathway (Dardari et al., 2025; Mesgari et al., 2022).

### 7.4 Marine antifouling and high-touch surfaces

Copper-based antifouling paints have been the marine industry standard since Tributyltin (TBT) prohibition (Dafforn et al., 2011). El Saeed et al. demonstrated that Cu_2_O NPs in epoxy provide equivalent biocidal performance to conventional Cu_2_O powder at lower total Cu loading (El Saeed et al., 2016). Kang et al. advanced the concept with Ti_3_C_2_T_x_@Cu-BTC@EP composites, simultaneously enhancing low-frequency impedance modulus 3.15-fold (corrosion protection) and achieving 100% *E. coli* lethality under light exposure within 24 h, addressing the co-existing biofouling and corrosion challenge on steel hulls (Kang et al., 2025). Thai et al. assessed fluoropolymer nanocomposite antifouling coatings on steel substrates in marine environments, demonstrating the role of nanoparticle dispersion quality in biofouling resistance (Thai et al., 2023), while Zhang et al. immobilised Cu-MOF within SiO_2_ aerogel matrices to extend antifouling coating longevity through controlled Cu^2+^ diffusion from the porous silica reservoir, an architectural approach directly applicable to transparent hard-surface coatings (Zhang et al., 2025). Hard surface high-touch environments (ATM interfaces, hospital touchscreens, elevator controls) represent the largest unmet commercial need and the lowest TRL (3–4): these surfaces simultaneously require >90% transmittance, extreme mechanical durability (thousands of daily contact events), and demonstrated *in situ* antimicrobial activity without external activation, the precise intersection of the transparency-durability-efficacy trilemma. No corpus system meets all three requirements.

The transition from conventional Cu_2_O antifouling paints to CuNM-enhanced formulations offers specific performance advantages beyond simple biocide equivalence. Nano-Cu_2_O particles demonstrate reduced Cu leaching rates at equivalent biocidal activity compared to coarse polycrystalline bulk Cu_2_O, a direct consequence of slower, matrix-controlled dissolution kinetics that extends functional coating lifetime before reapplication becomes necessary, directly reducing hull dry-dock operating costs (El Saeed et al., 2016). The MXene-enhanced Cu-BTC/epoxy composite of Kang et al. is the corpus’s most architecturally sophisticated marine system, combining the barrier corrosion-inhibiting properties of Ti_3_C_2_T_x_ MXene, Cu^2+^ biocidal function of Cu-BTC MOF, and mechanical durability of epoxy binder in a single coating layer (Kang et al., 2025). The measured 3.15-fold improvement in low-frequency impedance modulus (EIS) quantifies the corrosion protection contribution, a benchmark usually absent from marine CuNM publications that focus exclusively on biocidal performance. The fundamental commercial limitation of the MXene approach is cost: Ti_3_C_2_T_x_ is currently produced at USD 80–150/g at research scale, making hull-coat formulation economically implausible without substantial production scale-up; however, the architectural principle, high-performance nano-additive at <1 wt% augmenting a conventional industrial binder, is immediately applicable using more cost-accessible nanofillers at higher loadings (El Saeed et al., 2016; Gangadoo et al., 2020; Kang et al., 2025).

## 8 Translational considerations: scale-up, regulation, economics, and safety

### 8.1 Synthesis scale-up and manufacturing integration

Laboratory CuNM synthesis is well-developed at milligram-to-gram scale (Singh et al., 2023); the transition to kilogram-per-day continuous production introduces batch-to-batch variability through poor control of local temperature and concentration gradients in large reactors (Khalifa et al., 2025). Rai et al. represent the corpus’s clearest scale-up demonstration: gram-scale Cu_2_O synthesis by ascorbic acid reduction, coupled to dip-coat deposition on filter paper, necessary steps that are rarely linked in the literature (Rai, 2023). Green synthesis routes (plant extract or microbial metabolite reduction) offer potential environmental benefits, but characteristically broad size distributions and variable surface chemistry create unacceptable batch-to-batch variability at commodity scale (Kumeleh et al., 2026; Nayak et al., 2025). The key manufacturing integration advantage of CuNM coatings is their compatibility with existing industrial infrastructure: textile PDC finishing lines (30–100 m/min) and hard surface spray/roll-coat systems can accommodate CuNM suspensions as functional additives without capital-intensive equipment replacement (Karypidis et al., 2023; Singh et al., 2023). Critical unmet development steps include stable, shelf-life-compatible CuNM suspension formulations with rheology and surface tension specified for target deposition methods, not addressed in any corpus paper.

### 8.2 Regulatory landscape

The EU Biocidal Products Regulation (BPR 528/2012/EU) explicitly requires that nanoforms be assessed separately from bulk forms, an approval for conventional CuO does not extend to nano-CuO without additional nanoform characterisation data, substantially extending regulatory timelines and cost (Kang et al., 2025; Khalifa et al., 2025). EU Regulation 10/2011 imposes a 5 mg/kg Cu migration limit for food contact, as described in Section 7 (Men et al., 2025). For medical devices, both EU MDR 2017/745 and FDA 510(k)/*De Novo* pathways require full ISO 10993-1 biocompatibility datasets, with direct extrapolation from silver-coated device precedents not permissible given the distinct toxicological profile of Cu^2+^ vs. Ag^+^ (Bose et al., 2025; Tavakoli et al., 2019). EPA FIFRA registration for a novel antimicrobial surface claim typically requires 5–10 years and multi-million-dollar investment in efficacy, environmental fate, and human health data, the dominant economic barrier for high-touch surface applications.

### 8.3 Economic analysis and cost competitiveness

Economic viability is a first-order commercial consideration addressed quantitatively by only 44 of the 303 corpus papers with retrievable full text (14.5%), none of which reports a cost model spanning formulation development, stability testing, quality control and regulatory approval, yet it is the decisive factor in technology selection for industrial partners (Singh et al., 2023). CuNM raw material costs must be quoted on an explicit basis, since feedstock metal and finished nanopowder differ by one to two orders of magnitude. Copper metal traded at USD 13.4–14.2/kg (LME official close, August 2026) against approximately USD 2,117/kg for silver, giving a feedstock cost ratio close to 150-fold (London Bullion Market Association, 2026; London Metal Exchange, 2026) in copper’s favour. The figures previously reported here for Cu^0^ NPs, for Cu_2_O NPs and for silver nanoparticles all lay below the prevailing price of the corresponding metal and are therefore superseded. Conversion to nanoparticulate form adds a premium that is volume-dependent and rises by one to two orders of magnitude at research scale; equivalent conversion premia for silver are not publicly documented, so the finished-nanomaterial ratio cannot be stated with confidence and only the feedstock ratio is quoted here. It is this feedstock advantage, rather than any claim about finished nanopowder pricing, that determines long-term competitiveness in high-volume applications (Khalifa et al., 2025). For a textile functional finish at 1 wt% CuNM loading on 150 g/m^2^ fabric, the copper feedstock contributes 1.5 g Cu/m^2^, or approximately USD 0.02/m^2^ at current metal prices, before any cost of conversion to nanoparticulate form; the total reported material cost contribution is approximately USD 0.02–0.04/m^2^, well within the range of existing functional finishes (fluorocarbon DWR USD 0.05–0.08/m^2^; nanosilver USD 0.50–1.20/m^2^) (Khalifa et al., 2025). For transparent hard surface coatings requiring Cu_2_O quantum dots or CuS NPs at 0.1 wt% loading, material costs are higher (USD 0.10–0.25/m^2^ at current prices) but would decrease substantially at commodity production scale. The dominant economic barrier is not material cost but regulatory compliance, which must be separated into three components that the corpus consistently conflates. The first is the statutory registration service fee, which is documented and comparatively modest: under the US EPA PRIA 5 schedule for FY2025–2026, a new antimicrobial active ingredient for non-food use costs USD 292,592 with a 23-month statutory decision review, rising to USD 345,729 for direct food use over 26 months and falling to USD 120,734 for a low-risk non-food active over 14 months (Environmental Protection Agency, 2026); in the European Union, ECHA charges a EUR 10,000 notification fee, deductible from the approval application, with a Biocidal Products Committee opinion due within 270 days and Member State evaluation fees payable in addition (European Chemicals Agency, 2013). The second is the data-development package supporting the dossier, comprising toxicology, efficacy and environmental fate studies, and it is here that expenditure of the order of several million US dollars per active substance arises; this figure is an industry estimate rather than a published fee and should be treated as such. The third is elapsed commercial time, typically five to 10 years from formulation to market, which is governed by data generation and iteration rather than by the statutory review periods above. Beyond regulatory cost, a credible cost model for a CuNM coating must further account for formulation development, accelerated and real-time stability testing, batch-release quality control, and the scale-dependence of each; none of the 303 corpus papers with retrievable full text reports any of these categories (Supplementary Dataset S1, Sheet 9), and their absence, rather than any disagreement over material price, is the principal obstacle to economic assessment of this field. These costs must be judged against the addressable market of each sector rather than in absolute terms. Market research estimates place antimicrobial medical textiles at approximately USD 11.4 billion (2025, growing at about 7% annually) and antimicrobial medical device coatings approximately USD 3.0 billion (2026, about 14% annually), while estimates of the overall antimicrobial coatings market range from USD 6 billion to USD 17 billion for 2026 depending on the scope adopted by the reporting vendor (360iResearch, 2026; Sawal, 2026; Bidwai, 2026), a spread that itself counsels caution in the use of such figures. Against a multi-billion-dollar textile or medical device market, a registration fee of order USD 0.3 million and a data package of a few million dollars amortise readily across product volume; against the high-touch transparent surface segment, which remains at TRL 3–4 with no established product category, the same expenditure cannot presently be justified. This cost profile therefore favours large industrial entrants with existing regulatory infrastructure and argues for sector-focused commercialisation strategies that target highest-value first applications, wound care or healthcare textiles, before pursuing the more demanding and slower-to-approve high-touch transparent surface market (Rai, 2023; Singh et al., 2023).

### 8.4 Toxicity and environmental safety

The key safety concept is the therapeutic window: the Cu^2+^ concentration range achieving antimicrobial efficacy (0.5–5 mg/L against planktonic *S. aureus* and *E. coli*, 24-h contact) without cytotoxicity to mammalian cells (onset at ~50 mg/L for human cell lines). CuO NPs are consistently the most cytotoxic Cu form at equivalent mass loading (faster CuO dissolution to bioavailable Cu^2+^), followed by Cu_2_O, Cu^0^, CuS, and Cu-MOF, the same sequence as dissolution kinetics (Glazkova et al., 2022; Okoye et al., 2023; Reijnders, 2023; Tavakoli et al., 2019). *In vivo* wound healing studies (Chen et al., 2023; He et al., 2024; Sun et al., 2024) show no systemic toxicity at therapeutically relevant CuNM doses; however, formal toxicokinetic studies for dermal absorption from CuNM-coated textiles and environmental fate under realistic wash effluent scenarios are entirely absent from the corpus, the primary data gaps for regulatory dossier completion (Gutnik et al., 2026; Reijnders, 2023). Cu is a regulated aquatic contaminant (US EPA freshwater criterion 9 μg/L; Daphnia magna LC50 ~0.02–0.1 mg/L), and the scale of potential CuNM textile use warrants proactive environmental fate modelling, noting that speciation-based bioavailability assessment (biotic ligand model) typically yields more favourable risk characterisation than total-Cu metrics (El Saeed et al., 2016; Gangadoo et al., 2020).

## 9 Critical gaps and research outlook

### 9.1 Priority 1: resolving the transparency–durability–efficacy trilemma

The trilemma documented in this review arises because the relevant research communities (CAT3: durability, 16 articles; CAT9: transparency, 1 article; CAT4: antimicrobial mechanisms, 26 articles) have largely proceeded in parallel, each optimising a single metric independently. Convergent multi-objective research is the field’s highest priority. The most feasible near-term routes, in approximate order, are: (i) CuS NPs at sub-threshold visible loading with NIR photothermal supplementation; (ii) Cu_2_O quantum dot (<5 nm) systems exploiting quantum confinement to reduce visible absorption while maintaining surface area for dissolution; (iii) Cu-MOF hybrid systems (<0.5 wt% loading) combined with secondary antimicrobials (ZnO, QAC) at below-coloration Cu concentrations; and (iv) spatially heterogeneous coating architectures concentrating Cu in sub-visual-resolution micropatterns (Hu et al., 2022; Luo et al., 2025; Ran et al., 2021). Systematic design-of-experiment studies targeting simultaneous optimisation of Transparency (T), Efficacy (E), and Durability (D), not each independently, are urgently needed.

### 9.2 Priority 2: standardisation and the minimum reporting standard

The standardisation deficit identified throughout this review is the upstream bottleneck preventing evidence synthesis, regulatory assessment, and commercial investment decisions. The 8-parameter MRS proposed in this section is a concrete and immediately actionable first step requiring no new experimental work, only journal, author, and reviewer commitment to consistent reporting. Table 5 defines the eight mandatory reporting parameters, each grounded in a documented evidence gap identified in this corpus.

**TABLE 5:** Eight-parameter Minimum Reporting Standard (MRS) for CuNM antimicrobial coating studies. Each parameter addresses a documented evidence gap identified in this 498-article corpus.

Parameter	Evidence gap (documented in this review)	Mandatory reporting element
1. CuNM form and oxidation state	Cu^0^, Cu_2_O, CuO, CuS, and Cu-MOF differ in killing mechanism, ion release kinetics, and optical absorption (Section 2; Table 1); conflation prevents mechanism attribution (Khalifa et al., 2025; Pellosi et al., 2025)	XRD- or XPS-confirmed phase; specify primary and any secondary phases if mixed
2. Mean particle size and polydispersity index (PDI)	Size governs LSPR wavelength, specific surface area, and dissolution rate; quantum confinement below ~10 nm alters colour and potency (Section 2), yet 47% of the 303 corpus studies with retrievable full text report no numerical particle size (Supplementary Dataset S1, Sheet 10; Hu et al., 2022; Khalifa et al., 2025)	TEM number-mean diameter (nm) ± SD; DLS intensity-mean with PDI; state measurement medium
3. Cu loading and surface density	Loading reported in non-interconvertible units (wt%, mg/cm^2^, mg/L, mmol) across the corpus, preventing dose–response reconstruction (Sections 3–4; Chen et al., 2025a; El Saeed et al., 2016)	Report wt% in composite and mg Cu/cm^2^ on surface; state quantification method (ICP-OES or AAS after acid digestion)
4. Substrate type and surface pre-treatment	Pre-treatment omitted from majority of corpus fabrication studies yet is the primary determinant of wash durability; the same anchoring chemistry yields ≥40 cycles on pre-treated PET but <10 on untreated cotton (Section 3; Hu et al., 2022; Ran et al., 2021)	Specify substrate material; list all pre-treatment steps (degreasing agent, plasma conditions or acid etch, surface roughness Rz in µm if applicable)
5. Wash durability standard, detergent, and cycle count	AATCC 61, ISO 6330, and GB/T 12490-2014 use different temperatures, detergents, and mechanical actions; no validated inter-standard conversion factor exists in the corpus (Section 5; Karypidis et al., 2023; Singh et al., 2023)	State standard name and year (e.g., GB/T 12490-2014); detergent type; water temperature (°C); cycle count completed. For hard surfaces, state equivalent accelerated ageing protocol
6. Test organism, strain, and antimicrobial method	Zone-of-inhibition tests were used as the sole proxy for contact-kill in 36.7% of the textile corpus studies with retrievable full text (Supplementary Dataset S1, Sheet 6), overestimating surface efficacy; Gram-positive and Gram-negative organisms differ 3–5-fold in intrinsic Cu susceptibility (Section 7; Gangadoo et al., 2020; Karypidis et al., 2023).	State standard (ISO 20743, AATCC 100, or JIS L 1902 for textiles; ISO 22196 for hard surfaces); organism and strain number (e.g., *S. aureus* ATCC 6538); inoculum density (CFU/mL); contact time (h)
7. Quantitative efficacy metric (log-reduction)	% reduction compresses differences above 99% (99% = 2-log; 99.9% = 3-log); qualitative pass/fail ratings prevent cross-study meta-analysis and dose–response modelling (Karypidis et al., 2023; Singh et al., 2023)	Report log_10_ CFU reduction relative to untreated control ±SD; state detection limit (log CFU); report both immediate and extended time-point values for contact-kill studies
8. Optical transmittance at 550 nm	Treated as a design objective by only eight of 498 corpus articles (1.6%), with three reporting a quantitative transmittance value, despite determining commercial viability for all transparency-critical applications (touchscreens, food packaging windows, medical housings) (Section 6; Khalifa et al., 2025; Pellosi et al., 2025)	Report luminous transmittance at 550 nm and haze (%) by UV-Vis spectrophotometry per ASTM D1003, on the coated substrate versus an uncoated control; state film thickness (µm) and substrate transmittance baseline. Transmittance alone is insufficient, since a coating may exceed 80% transmittance yet appear milky through scattering

Adoption of Table 5 by leading journals (ACS Nano, Advanced Materials, Journal of Hazardous Materials, Applied Surface Science, Carbohydrate Polymers) as a mandatory submission checklist would generate a structured evidence base for the next-generation of meta-analyses and ML-driven design within 3–5 years. Alongside the MRS, validated accelerated ageing protocols, standardised thermal cycling, UV irradiation, chemical exposure, and mechanical stress sequences that predictably reproduce real-world service ageing are needed to replace the current reliance on short-term tests extrapolated without validation (Karypidis et al., 2023; Khalifa et al., 2025; Singh et al., 2023).

### 9.3 Priority 3: smart coatings, virucidal testing, and AI-accelerated design

Next-generation smart CuNM coatings should integrate: (i) infection-sensing elements (pH, temperature, bacterial metabolites); (ii) stimulus-responsive Cu^2+^ release triggered by infection signals (already demonstrated by (Bi et al., 2026; Kruk et al., 2019)); (iii) self-healing matrix components to address surface damage; and (iv) rechargeable Cu-MOF architectures for reservoir replenishment without recoating, a concept demonstrated only at milligram scale in water treatment membranes and requiring translation to surface coating contexts (Hubab et al., 2025; Kang et al., 2025). Virucidal performance remains critically understudied: of the 303 corpus papers with retrievable full text, 29 (9.6%) quantify antiviral activity, and the single most relevant study ((Foffa et al., 2022), >99% SARS-CoV-2 inactivation) tests only one CuNM formulation against one enveloped virus; more recently, Bakhet et al. demonstrated DLC:Cu thin films achieving up to 2.23-log coronavirus and 1.63-log herpesvirus reductions after 1-h contact (Bakhet et al., 2024), while Bakhet et al. confirmed equivalent virucidal efficacy for laser-generated CuNP coatings against both RNA (coronavirus) and DNA (herpesvirus) model viruses (Bakhet et al., 2025). Systematic virucidal testing across CuNM forms, enveloped and non-enveloped viruses, and contact time-response curves is a key recommendation.

### 9.4 Priority 4: environmental fate, life-cycle assessment, and responsible scaling

The environmental profile of CuNM coating deployment at commercial scale requires proactive assessment before the market reaches levels generating measurable aquatic or terrestrial Cu loading. CuNM textile wash effluents are the most quantified exposure pathway: a single 30 °C domestic wash of a CuNP-finished textile can release 5%–15% of surface Cu loading per cycle (Singh et al., 2023), translating to 0.5–2 mg Cu per garment per wash under conventional loading conditions. For a hypothetical 10% market penetration scenario in the EU antimicrobial textile market, this represents approximately 300–800 kg additional Cu per day entering urban wastewater treatment networks, a tractable burden for modern activated sludge systems (>95% Cu removal efficiency) but potentially significant for ageing treatment infrastructure in middle-income countries. More critically, Cu concentrates in sewage sludge applied as agricultural fertiliser, creating a secondary terrestrial pathway whose long-term consequences have not been assessed for CuNM specifically. Life-cycle assessment studies comparing CuNM antimicrobial textiles against AgNP equivalents or conventional quaternary ammonium finishes across production–use–end-of-life are entirely absent from the corpus. Carbon footprint, mineral depletion potential, and freshwater ecotoxicity potential throughout the value chain have not been quantified for any CuNM coating system, a gap that is simultaneously a regulatory prerequisite under both EU BPR and EPA FIFRA and an opportunity to demonstrate the environmental advantages of lower-dose, higher-efficiency CuNM systems over higher-loading bulk Cu approaches (El Saeed et al., 2016; Gangadoo et al., 2020; Men et al., 2025).

## 10 Conclusion

This review synthesises 498 peer-reviewed articles on CuNM antimicrobial coatings to address the durability–transparency–efficacy trilemma that has prevented translation of impressive laboratory performance into commercial deployment. Five principal conclusions emerge with high confidence. First, CuNMs in all five principal forms (Cu^0^, Cu_2_O, CuO, CuS, Cu-MOF) demonstrate broad-spectrum antimicrobial efficacy against Gram-positive and Gram-negative bacteria, fungi, and enveloped viruses including SARS-CoV-2. Multiple simultaneous killing mechanisms (Cu^2+^ membrane disruption, ROS generation, photothermal hyperthermia) make resistance development intrinsically unlikely, and the approximately 150-fold feedstock cost advantage over silver makes CuNM coatings commercially compelling. Second, coating durability has advanced substantially through catechol-based anchoring chemistry, polydopamine-mediated GO/Cu_2_O attachment to PET (40 laundering cycles (GB/T 12490-2014) at 90% efficacy) and core-shell CAT-TEPA encapsulation of Cu_2_O NPs, establishing proof-of-concept architectures that address both mechanical detachment and oxidation state drift simultaneously. Third, ion release engineering has progressed from passive burst-release toward stimulus-responsive selectivity: infection-responsive pH-triggered systems and NIR-photothermal CuS platforms extend the functional lifetime of the Cu reservoir while reducing unnecessary host cell exposure in sterile conditions. Fourth, and most critically, no published CuNM coating simultaneously demonstrates ≥80% transmittance, ≥99% bacterial reduction, and ≥50 home laundering or ≥3-month durability, and this conclusion is insensitive to reasonable variation in the three thresholds. This trilemma is the field’s defining unresolved challenge and the primary commercial barrier for the highest-value applications, transparent hard surfaces, touchscreens, and high-performance food packaging. Fifth, the absence of standardised reporting across the literature, different wash standards, efficacy metrics, organisms, and time points, makes quantitative evidence synthesis and regulatory assessment currently impossible. The Minimum Reporting Standard proposed here (Section 9) is a concrete, zero-cost first step toward resolving this deficit.
